# Allergic Bronchopulmonary Aspergillosis (ABPA) Without Asthma: A Case Report and Review of the ABPA Imaging Spectrum

**DOI:** 10.7759/cureus.97250

**Published:** 2025-11-19

**Authors:** Sheikh Jamal, Abdulwahab Zabara, M Josheel Naveed, Muhammad Yousaf, Hatem M Abusriwil, Farah J N Assaf, Bassem Al Hariri

**Affiliations:** 1 Medicine, Hamad Medical Corporation, Doha, QAT; 2 Clinical Imaging, Hamad General Hospital, Doha, QAT; 3 Medicine, Weill Cornell Medicine Qatar, Doha, QAT; 4 Pulmonology, Hamad Medical Corporation, Doha, QAT; 5 Internal Medicine, Hamad Medical Corporation, Doha, QAT

**Keywords:** abpa, abpa imaging, abpa radiology overview, allergic bronchopulmonary aspergillosis(abpa), allergy, asthma, lymphadenopathy

## Abstract

Allergic bronchopulmonary aspergillosis (ABPA) is typically associated with underlying asthma or cystic fibrosis, and its diagnosis is often based on a combination of clinical, immunological, and radiological findings. We report a case of ABPA in a patient without a prior diagnosis of asthma. Clinical suspicion and subsequent tests for ABPA are usually requested by physicians for poorly controlled asthma patients or cases of recurrent asthma attacks. Due to the non-specificity of symptoms and, often, radiology findings, the absence of a history of asthma lowers suspicion for ABPA, potentially resulting in diagnostic delays. This usually leads to over-investigation, including invasive procedures, to explore other potential diagnoses adequately. There have been case reports of ABPA diagnosed in non-asthmatic patients. Considering our case and previous similar cases, we propose a reconsideration of diagnostic criteria to encompass nonasthmatic patients displaying typical clinical, biochemical, and radiological features of ABPA.

## Introduction

*Aspergillus fumigatus* and other *Aspergillus* species are ubiquitous. *Aspergillus *infections can present in diverse forms. Allergic bronchopulmonary aspergillosis (ABPA) affects approximately 13% of patients attending asthma clinics [1]. The International Society for Human and Animal Mycology (ISHAM) identifies asthma and cystic fibrosis (CF) as key predisposing conditions in its diagnostic criteria [2]. ABPA represents one manifestation of *Aspergillus *infection, characterized by a complex hypersensitivity reaction initiated by the colonization of the airways with *Aspergillus *fumigatus. In chronic cases, recurrent episodes of bronchial obstruction, inflammation, and mucoid impaction may result in bronchiectasis, fibrosis, and compromised respiratory function [3]. Its diagnosis is based on clinical features, laboratory investigations including peripheral eosinophilia, and a wide range of observations on diagnostic imaging. Usually, there is a history of asthma or CF, and hence the European Respiratory Society and ISHAM mention a history of asthma as one of the diagnostic criteria for ABPA. We present a case of ABPA that presented a diagnostic challenge as the patient lacked a history of asthma. Extensive assessments and investigations, including endobronchial ultrasound-guided transbronchial needle aspiration (EBUS-TBNA), bronchoscopy, and bronchial washings, did not confirm a diagnosis, and ultimately the patient required an image-guided lung biopsy for confirmation.

## Case presentation

A 39-year-old Bangladeshi male patient, who was a driver and a lifelong non-smoker, presented to the pulmonology clinic with a two-week history of productive cough and intermittent fever. He denied any weight loss or hemoptysis. Six months before his presentation, he was admitted to the hospital and treated for pneumonia. His symptoms and chest X-ray findings had almost completely resolved during a follow-up visit to the pulmonology outpatient department. Besides a prior history of pneumonia, he had no other history of pulmonary disease or occupational dust exposure.

On physical examination, the patient appeared comfortable at rest with no signs of respiratory distress or shortness of breath. His temperature was 36.6 degrees°C, respiratory rate was 19 breaths per minute, and oxygen saturation was 99% on room air. His body mass index (BMI) was 29.36 kg/m². There was no digital clubbing or supraclavicular lymphadenopathy. His chest was clear on auscultation.

**Figure 1:**
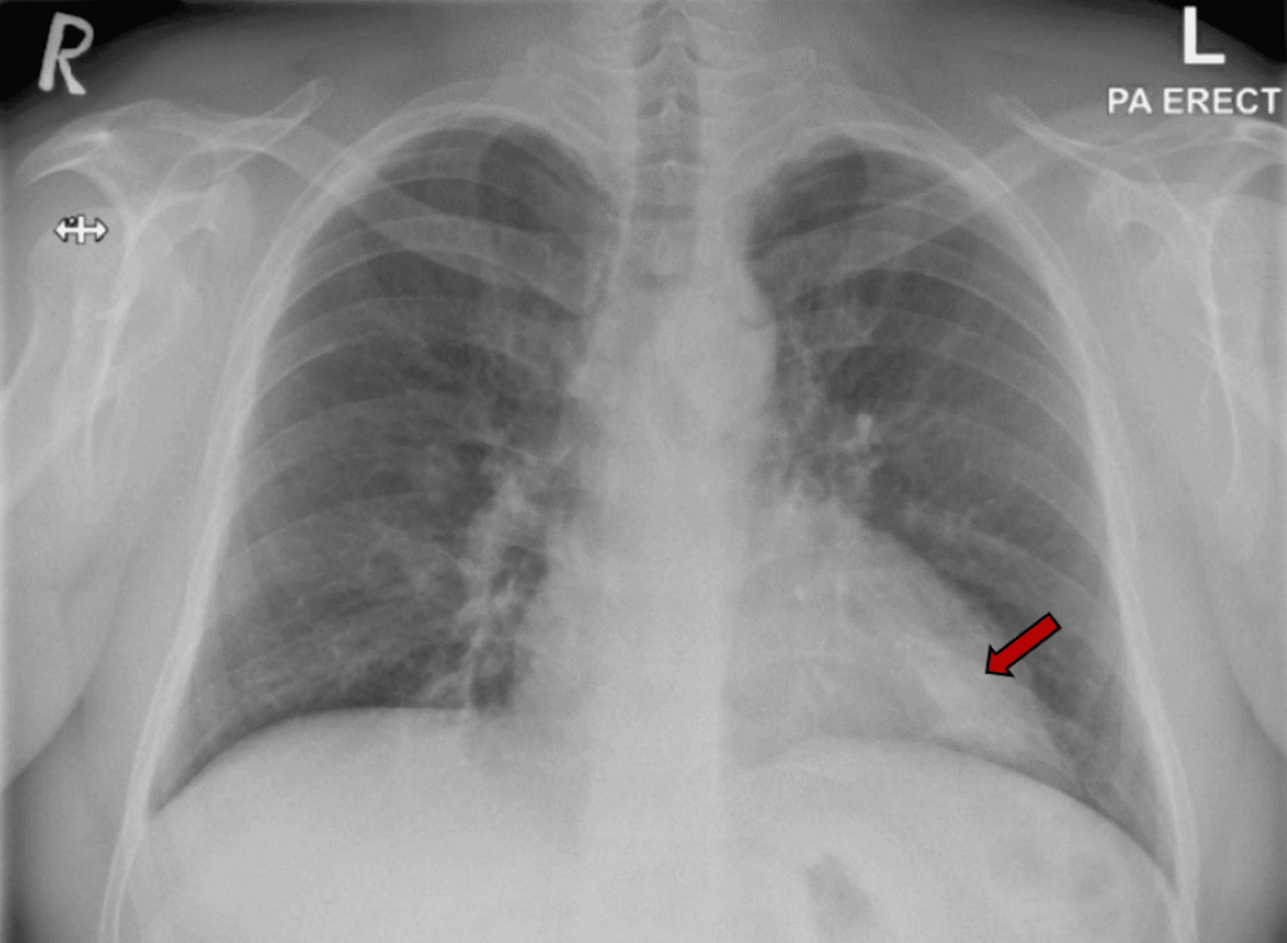
The posteroanterior chest radiograph shows a left retrocardiac patchy consolidation (arrow).

**Figure 2:**
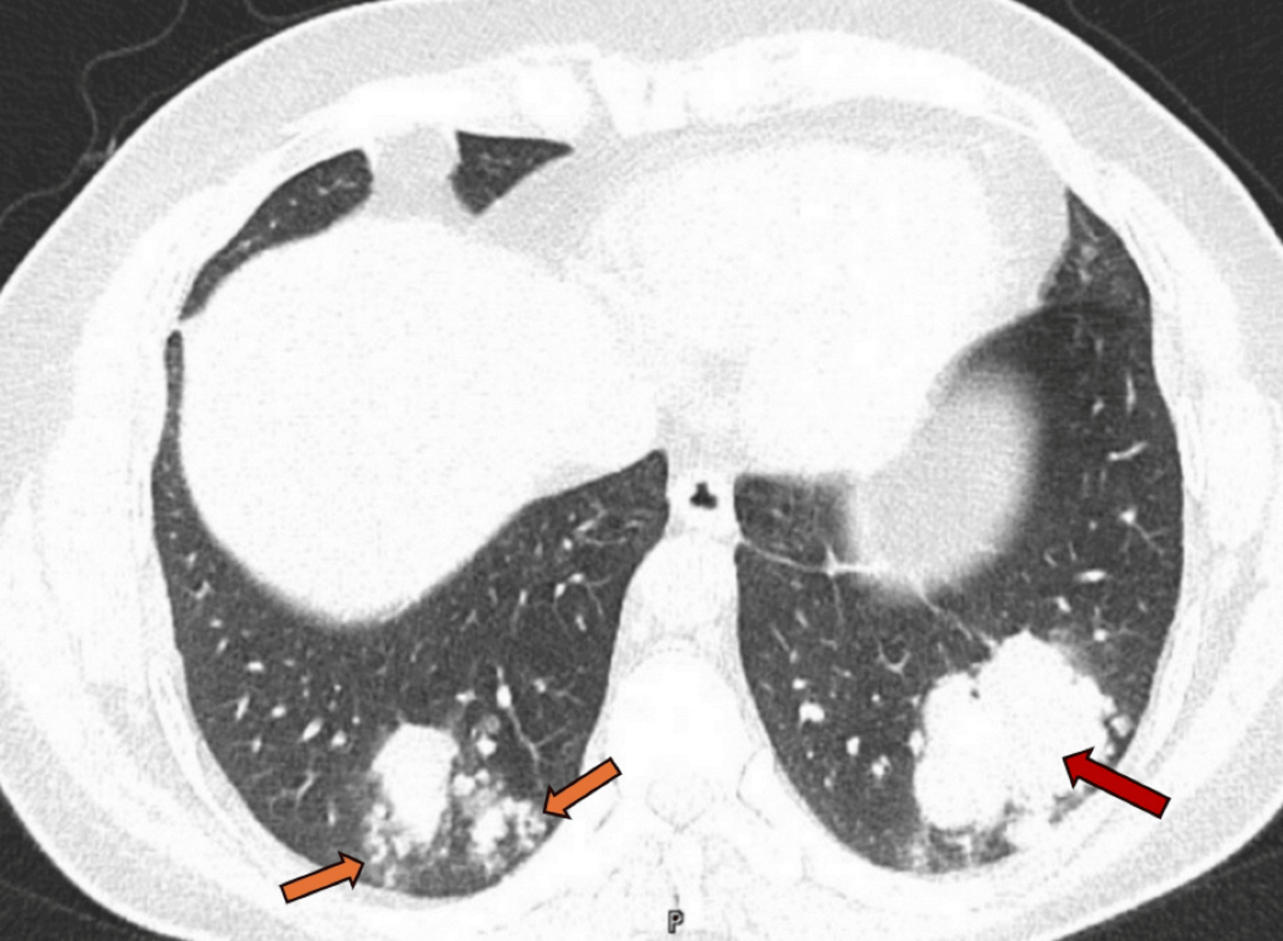
A CT scan of the chest (axial image in the lung window) shows bilateral basal mass-like lobulated opacities. Small nodular opacities are also seen surrounding the lesions (orange arrows). A biopsy was obtained from the large lesion in the left lower lobe (red arrow).

**Figure 3:**
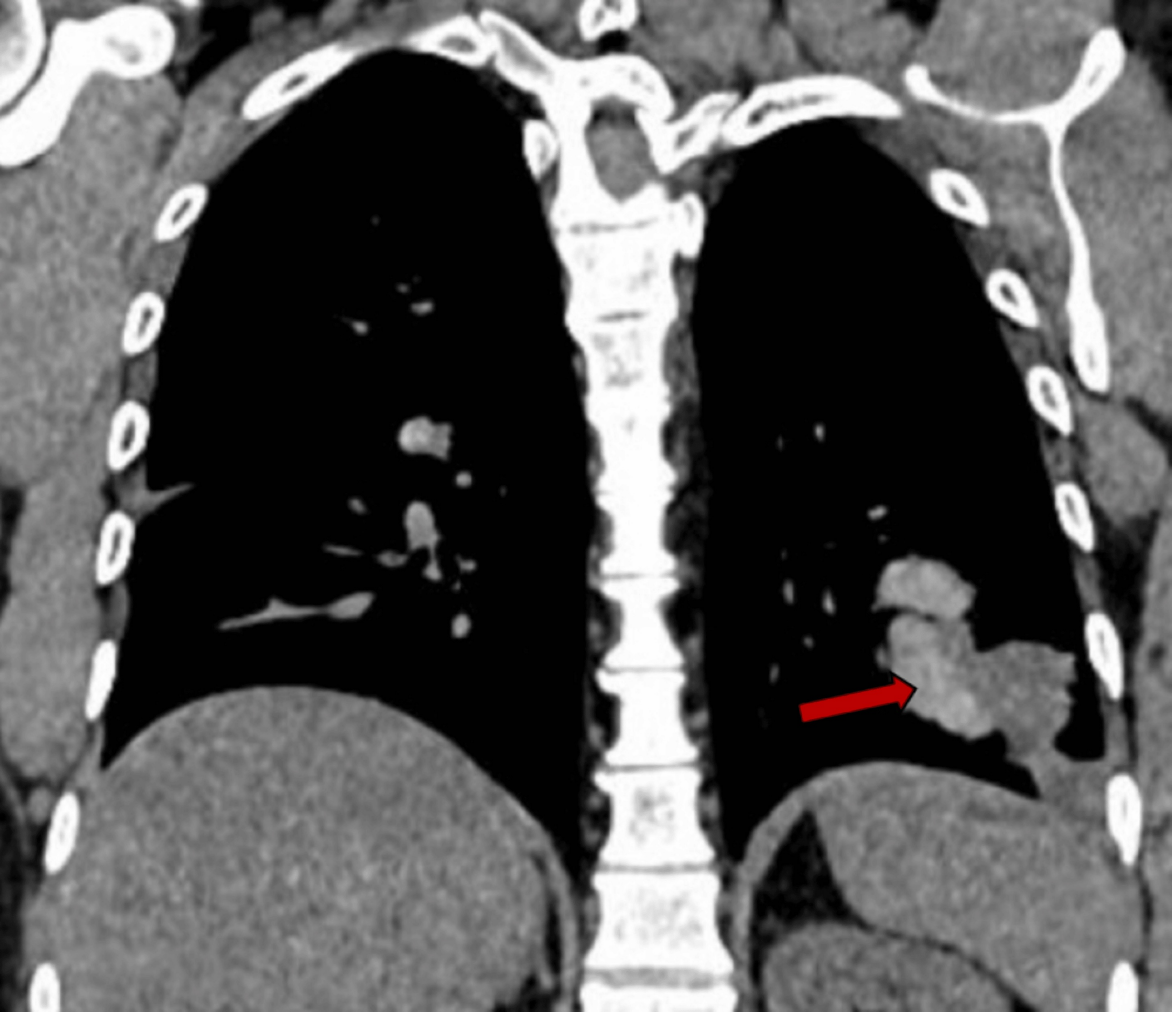
The CT of the chest (coronal, mediastinal window) demonstrates elongated, branching mucoid impaction within dilated bronchi of the left lower lobe. While not a classic “finger-in-glove” pattern, this represents a variant appearance of mucus-impacted bronchiectatic airways, typical of allergic bronchopulmonary aspergillosis, including a focus of high-attenuation mucus (red arrow).

**Figure 4:**
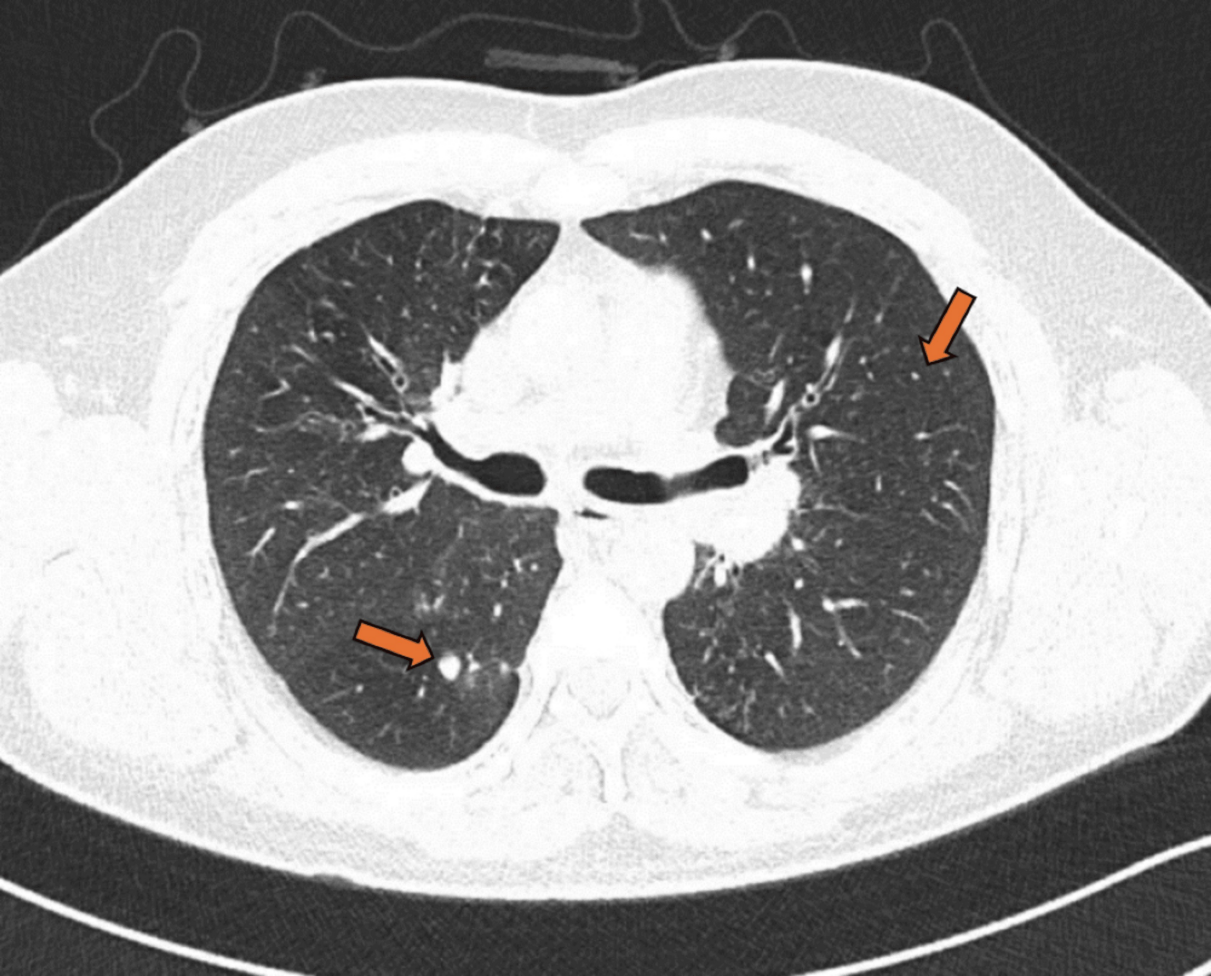
Bilateral scattered nodular opacities of varying size are noted (arrows).

**Figure 5:**
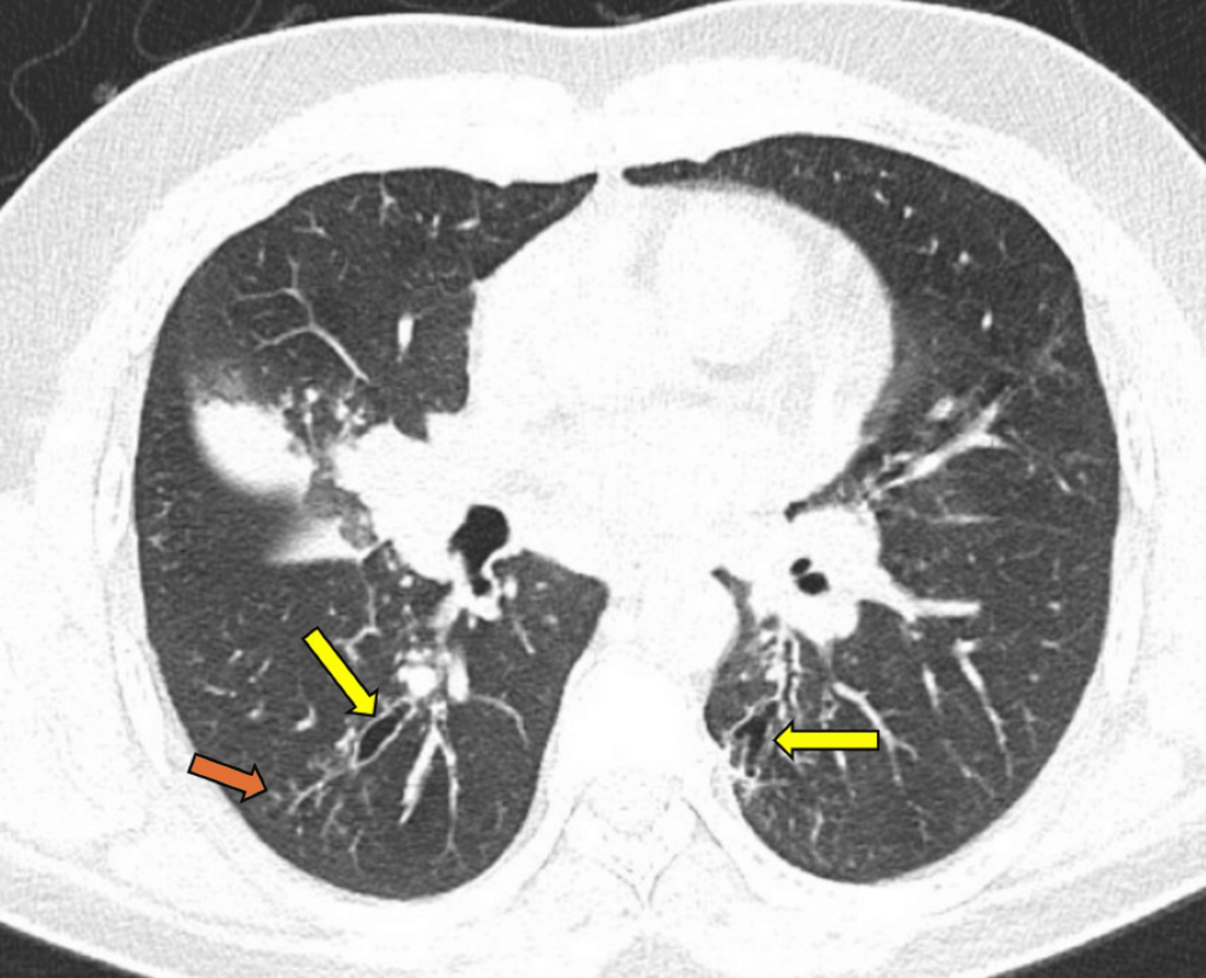
The axial image of the chest CT in the lung window demonstrates bilateral bronchiectasis (yellow arrows), along with small nodular opacities and tree-in-bud changes (orange arrow).

**Figure 6:**
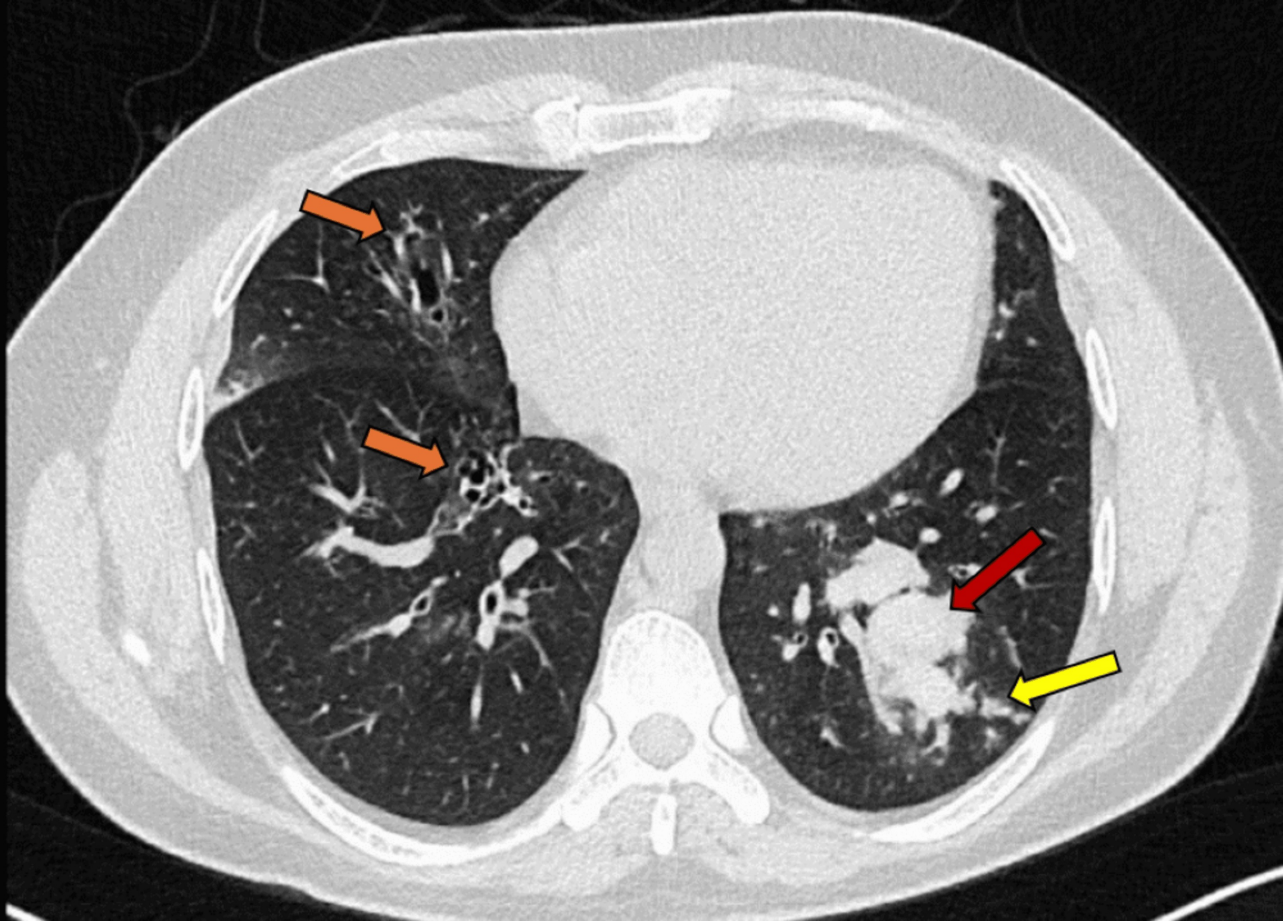
The axial image of the chest CT in the lung window shows bilateral central and peripheral bronchiectasis (orange arrows), as well as a mass-like lesion in the left lower lobe (red arrow) with adjacent small nodular opacities (yellow arrow).

**Figure 7:**
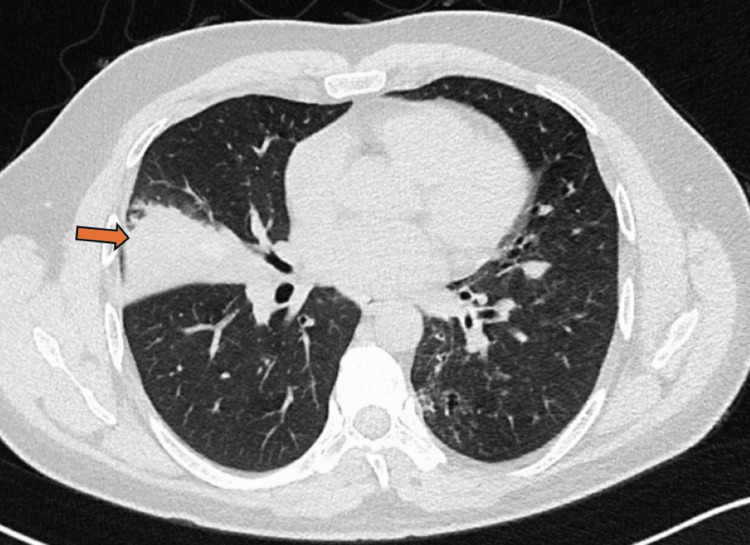
The axial image of the chest in the lung window shows collapse of the lateral segment of the right middle lobe (arrow).

**Figure 8:**
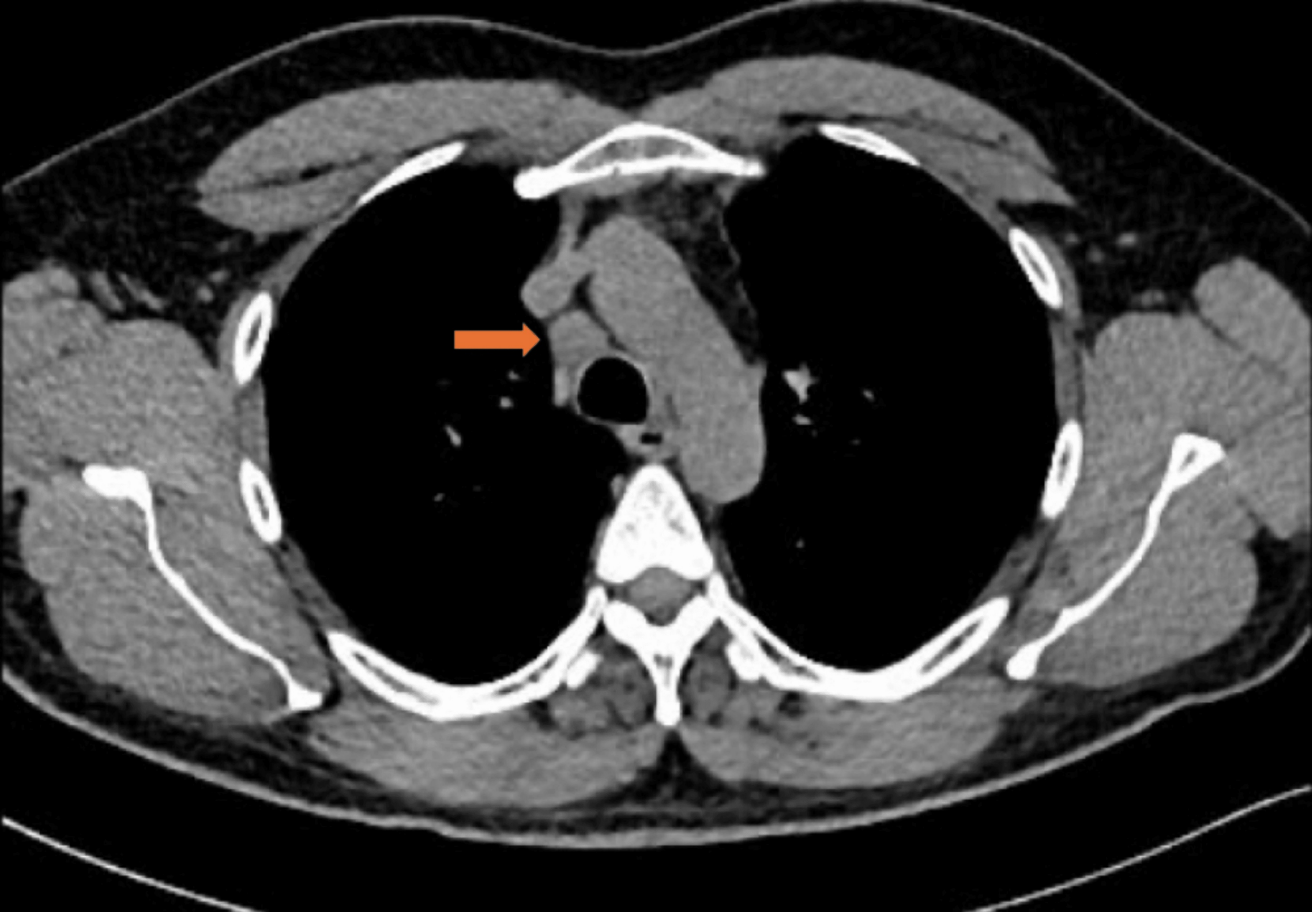
The axial image of the chest CT in the mediastinal window shows enlarged mediastinal (right lower paratracheal, station 4R) lymph node (arrow).

His chest X-ray indicated left retrocardiac patchy consolidation (Figure 1), which was not present on the previous radiograph. He was prescribed antibiotics, and given the recurrent pneumonia, a computed tomography (CT) scan of the chest was requested. His high-resolution chest CT scan revealed multiple abnormal findings. Bilateral lower lobe lobulated, elongated, branching mass-like lesions with adjacent small, ill-defined nodules were evident (Figures 2, 3). Additionally, bilateral scattered nodular opacities (Figure 4) and scattered peri-lesional tree-in-bud changes were observed. Multifocal bronchiectasis was noted both centrally and peripherally, exhibiting primarily two patterns: cylindrical and cystic bronchiectasis (Figures 5, 6). The scan also depicted the collapse of the lateral segment of the right middle lobe (Figure 7). Furthermore, enlarged bilateral hilar and mediastinal lymph nodes were present, with the largest measuring approximately 16 x 11 mm at the right lower paratracheal location (Figure 8).

His laboratory tests revealed a white blood cell count of 9.4 x 10³ µL, a hemoglobin level of 14.1 g/dL, and an eosinophil count of 1.8 x 10³/µL. His C-reactive protein was less than 2 mg/L. The antinuclear antibody, connective tissue disease screen, and antineutrophil cytoplasmic antibody were negative, while anti-cyclic citrullinated peptide antibodies were less than 8 units per milliliter (U/mL). Additionally, antibodies for *Echinococcus *and *Schistosoma *were negative. His total immunoglobulin E (IgE) level exceeded 10,000 kU/L. His specific IgE testing for mixed molds (MX2) and *Aspergillus fumigatus* (M3) showed positive results (M3 *Aspergillus fumigatus* was 17.6 kU/L, indicative of a class 4 high positive result). His sweat chloride test was normal.

His spirometry revealed a mild restrictive pattern, with a forced expiratory volume in 1 second (FEV₁) of 2.59 liters (L) (77% predicted), a forced vital capacity (FVC) of 2.95 L (74% predicted), and an FEV₁/FVC ratio of 0.88. Post-bronchodilation, there was no improvement in FEV₁. Given his high BMI, this was likely at least partially contributing to his mild restrictive pattern.

He underwent bronchoscopy and bronchial wash, which revealed the growth of *Pseudomonas aeruginosa*; however, the tuberculosis (TB) workup yielded negative results, and no fungal elements were identified. Given the high incidence of TB in the region and its inclusion among the differentials, he also underwent endobronchial ultrasound-guided transbronchial needle aspiration (EBUS-TBNA). The EBUS revealed mediastinal and right hilar lymphadenopathy, but the TBNA histopathology results did not reveal granulomas or malignancy.

Although his CT chest findings and laboratory tests suggested ABPA, the absence of a past history of asthma prompted further confirmation of the diagnosis. Consequently, he underwent a CT-guided lung biopsy. The histopathological analysis demonstrated consolidated lung parenchyma with dense chronic inflammatory infiltrates, particularly around the airways, and notably rich in eosinophils. The inflammation extended into the alveolar septa and perivascular regions, accompanied by necrotizing granulomas within the airspaces and prominent organizing pneumonia, but without evidence of established fibrosis. There was no neutrophilic inflammation, vasculitis, or evidence of malignancy. Although fungal stains were negative, this did not exclude a hypersensitivity reaction to fungi. Overall, the histological pattern was most suggestive of bronchocentric granulomatosis consistent with ABPA, particularly in the absence of features supporting alternative eosinophilic or granulomatous diseases such as eosinophilic pneumonia, eosinophilic granulomatosis with polyangiitis (Churg-Strauss syndrome), or granulomatosis with polyangiitis (Wegener's granulomatosis). These histological features aligned with the patient's clinical profile and radiological findings, confirming the diagnosis of ABPA.

Subsequently, he was commenced on treatment for ABPA in July 2022 with itraconazole 200 mg twice daily for four months and prednisolone, initially at 40 mg once daily, which was gradually tapered off over the following four months. His symptoms significantly improved following the initiation of treatment, and he demonstrated continuous clinical improvement during follow-up visits. Moreover, his repeat chest X-ray showed almost complete resolution of the opacity noted on his previous X-ray. The therapeutic regimen also led to a marked improvement in laboratory markers. Total serum IgE decreased significantly from 10,817 kU/L on June 6, 2022, to 1,681 kU/L by December 12, 2022, and further down to 1,425 kU/L in February 2023. Similarly, the peripheral eosinophil count dropped from 2.1 x 10⁹/L in June 2022 to 0.18 x 10⁹/L in August 2022, remaining low at 0.51 x 10⁹/L in December 2022. These findings reflected a robust immunologic response to the treatment.

## Discussion

ABPA represents one manifestation of *Aspergillus *infection, characterized by a complex hypersensitivity reaction initiated by the colonization of the airways with *Aspergillus fumigatus*. *Aspergillus*-related pulmonary diseases encompass a broad clinical spectrum. A simple aspergilloma represents a fungal ball developing within a pre-existing pulmonary cavity, often secondary to tuberculosis or sarcoidosis, and typically presents with hemoptysis. Chronic pulmonary aspergillosis is a slowly progressive infection seen in patients with underlying structural lung disease or mild immunosuppression, usually presenting with chronic cavitary lesions and systemic symptoms. Invasive pulmonary aspergillosis occurs primarily in immunocompromised individuals and is characterized by tissue and vascular invasion leading to necrotizing pneumonia. In contrast, ABPA, as demonstrated in our case, represents an immunologic hypersensitivity reaction to airway colonization by *Aspergillus fumigatus*, resulting in bronchial inflammation, mucoid impaction, and bronchiectasis.

Clinical suspicion of ABPA usually arises in patients who have been previously diagnosed with asthma or CF. Poor control of these conditions or recurrent exacerbations prompts physicians to investigate ABPA as the potential cause of overall poor disease control. In cases where there is no history of asthma or CF, the diagnosis of ABPA can be particularly challenging, as exemplified in our case. Asthma and CF have been mentioned as predisposing factors and a part of the diagnostic criteria in most ABPA guidelines. The ISHAM working group mentions asthma and CF as predisposing conditions in their diagnostic criteria. Additionally, their obligatory criteria include a total IgE of greater than 1000 international units per milliliter (IU/mL) and elevated IgE levels against *Aspergillus fumigatus* or a positive *Aspergillus* skin prick test [2]. Both of these should be present as part of the diagnostic criteria. Other criteria parameters, two out of three of which should be present, include raised precipitating or IgG antibodies against *Aspergillus fumigatus* in serum, eosinophils of greater than 500 cells per microliter (cells/µL), and radiological features consistent with ABPA [2]. Consequently, diagnosing ABPA in patients with established asthma or CF diagnoses is arguably less complex compared to individuals without any prior history of these conditions.

Our patient did not have a history of asthma; however, he fulfilled the rest of the diagnostic criteria (other than *Aspergillus precipitins*, which could not be checked). In individuals lacking a prior asthma diagnosis, it is conceivable that some may have had undiagnosed asthma until significant ABPA symptoms emerged. Our patient had no prior asthma diagnosis. Furthermore, his spirometry showed only a mild restrictive pattern without any increase in FEV₁ post-bronchodilation. His detailed medical history also did not indicate asthma during childhood or later in life. Despite the significant symptomatic overlap between different respiratory conditions and specifically between asthma and ABPA, we tentatively concluded that the patient had ABPA without underlying asthma, considering his medical history, spirometry results, and clinical response.

The mild restrictive pattern observed on spirometry in our patient likely resulted from a combination of contributory factors. First, the chest CT demonstrated collapse of the lateral segment of the right middle lobe, which would lead to segmental volume loss and a corresponding restrictive ventilatory defect. Second, the patient's high BMI of 31 kg/m² suggested that obesity-related restriction may have partially contributed by reducing chest wall compliance and diaphragmatic movement. Third, widespread mucus plugging, including the presence of high-attenuation mucus (HAM) and extensive bronchiectatic changes, could have caused localized compression of adjacent lung parenchyma or interfered with normal expansion. Finally, although pulmonary fibrosis was not confirmed histologically, chronic airway inflammation and structural remodeling in ABPA may contribute to restrictive physiology. Therefore, the observed spirometric pattern likely reflects the interplay of multiple structural and mechanical factors. Full pulmonary function testing, including lung volumes and diffusing capacity of the lung for carbon monoxide, would have provided additional information; however, this was not performed.

There have been documented case reports of ABPA occurring without a preceding diagnosis of asthma [4,5,6]. In the absence of asthma, patients often undergo investigations for other pulmonary conditions, notably TB and cancer. In our patient's case, a TB diagnosis remained under consideration due to its presentation, radiology findings including hilar and mediastinal lymphadenopathy, and the relatively high regional incidence rate of TB. This consideration prompted investigations, including EBUS-TBNA, which might not have been considered if the patient had a history of asthma or CF. Ultimately, a definitive diagnosis required tissue confirmation achieved through an image-guided lung biopsy. Our case exhibited mediastinal and hilar lymphadenopathy, which is rarely reported in ABPA. This is likely a consequence of the immune response.

Given the central role of imaging in identifying and characterizing ABPA, a concise overview of its typical radiologic manifestations is presented below to contextualize our case within the broader imaging spectrum of the disease. Radiologic imaging plays a pivotal role in the diagnosis, staging, and longitudinal assessment of ABPA, a hypersensitivity reaction to Aspergillus fumigatus colonizing the airways. The imaging features reflect the underlying processes of airway inflammation, mucus impaction, and structural bronchial wall damage, which evolve from reversible obstruction to chronic bronchiectatic changes and fibrosis. Chest radiography often provides the first diagnostic clue, showing transient, patchy, or migratory pulmonary opacities that correspond to mucus-filled bronchi. These "fleeting shadows" may shift in location over time. Mucus plugging of dilated bronchi can produce rounded or branching mass-like opacities, sometimes mistaken for consolidation or neoplasms. Characteristic radiographic signs include tram-line shadows (parallel bronchial walls seen longitudinally), ring shadows (cross-sectional bronchial dilatation), and toothpaste or finger-in-glove opacities caused by mucoid impaction. Atelectasis, usually subsegmental or segmental, may result from bronchial obstruction, occasionally progressing to lobar collapse. Importantly, these findings often resolve or migrate, aiding distinction from chronic infection or malignancy.

High-resolution CT of the chest provides a more detailed view and remains the gold standard for assessing disease extent and activity. The hallmark finding is central bronchiectasis, typically involving segmental and subsegmental bronchi with relative peripheral sparing, although a central-plus-peripheral distribution may also occur. Mucoid impaction within dilated bronchi produces the classical "finger-in-glove" appearance, whereas denser mucus collections may appear as "toothpaste shadows" or "Y/V-shaped opacities." The "tram-track sign" reflects thickened bronchial walls, while "ring shadows" denote cross-sectional views of bronchiectatic bronchi. The attenuation of mucus varies; most are low density, but in 20%-30% of cases, it appears hyperattenuating on CT, known as HAM, a relatively specific feature of ABPA and a marker of active disease [7,8]. Additional parenchymal findings include ground-glass opacities, consolidation, centrilobular nodules, tree-in-bud patterns, and mosaic attenuation from air trapping, indicating small-airway involvement. Chronic disease can progress to fibrotic remodeling, volume loss, and architectural distortion. Although uncommon, mediastinal or hilar lymphadenopathy may occur as part of the immune response. Radiologic interpretation, therefore, not only aids diagnosis but also helps monitor disease activity, guide therapy, and identify irreversible structural damage. The diverse spectrum of radiological findings observed in ABPA is summarized in Table 1, which provides a concise overview of the characteristic features on both chest radiograph and CT (Table 1).

**Table 1:** Radiology findings of allergic bronchopulmonary aspergillosis * Atelectasis is usually subsegmental or segmental, occasionally lobar, and rarely can involve an entire lung [7]. ** The bronchial mucus plugging in ABPA is generally hypodense but may also have high CT attenuation values [2, 8], also known as high attenuation mucous (HAM); *** Lymphadenopathy is rarely reported in ABPA. Our patient was found to have hilar and mediastinal lymphadenopathy [7].

Chest radiograph findings
Transient patchy consolidation
Mucous plugging of dilated bronchi and distal accumulation of secretions may appear as mass lesions. Similarly, large bronchoceles may appear as mass-like lesions.
Findings of bronchiectasis (e.g, “Tramline shadows”, “ring shadows” and “toothpaste shadows”. See below).
Subsegmental, segmental, lobar, or lung collapse as a result of mucoid impaction*.
Chest CT findings
Pulmonary parenchymal infiltrates and opacities.
A wide variety of pulmonary opacities are seen, including:
- Consolidation
- Ground glass changes
- Tree in bud changes and centrilobular nodules
- Scattered nodular opacities
Central or central and peripheral bronchiectasis
Tramline shadows” or “tram track sign”, indicative of parallel linear shadows reflecting the longitudinal views of thickened bronchial walls.
“Finger in glove” opacity: Indicative of mucoid impaction in dilated bronchi.
Toothpaste shadows (V, inverted V, Y shadows)”: Like “finger and glove” opacities, it is also indicative of mucoid impaction of the bronchi.
“Ring shadows”: Reflect dilated bronchi with thickened and inflamed bronchial walls.
Subsegmental, segmental, lobar, or lung collapse*.
Mucous plugging**, especially in larger airways, may be seen as mass lesions.
Bronchololes with focal bronchial dilation and mucoid accumulation may appear as mass-like lesions.
Mosaic attenuation: may appear due to air trapping.
Mediastinal or hilar lymphadenopathy may occur as part of the immune response***.
Chronic disease may progress to pulmonary fibrosis.

Similar cases to ours without a prior asthma diagnosis have significant implications in areas with a high prevalence of TB. Patients exhibiting such symptoms and radiology findings are often investigated for and, at times, empirically treated for TB [6]. Likewise, with similar presentations, cancer diagnosis remains a genuine concern for physicians. Berkin et al. [4], in their case series, mention their non-asthmatic patients having signs and symptoms highly suggestive of bronchial carcinoma before eventually being diagnosed with ABPA. Considering our case and previous similar cases, we propose a reconsideration of diagnostic criteria, encompassing non-asthmatic patients displaying typical clinical, biochemical, and radiological features of ABPA. In the absence of a history of asthma, many such patients may undergo investigations aimed at diagnosing cancer or TB, sometimes resulting in empirical TB treatment. Our patient also underwent invasive procedures, including EBUS-TBNA and a CT-guided lung biopsy, before confirming the diagnosis.

While the possibility of dual pathologies, such as ABPA coexisting with lung cancer or TB, must be acknowledged, diagnosing ABPA based solely on fulfilling the remaining diagnostic criteria, apart from a history of asthma or CF, with appropriate follow-up, may prove beneficial. This approach not only alleviates anxiety and psychological implications for the patient concerning other potential serious diagnoses, including lung cancer, but also appropriately reduces the necessity for a battery of invasive investigations and the associated costs.

## Conclusions

This case highlights the difficulty of diagnosing ABPA in patients without a previous history of asthma, as well as the complex task of balancing and determining the appropriate extent of diagnostic investigations, including invasive procedures, to adequately explore other potential diagnoses in such cases. In light of our case and comparable precedents, we suggest a reassessment of diagnostic protocols to incorporate nonasthmatic patients presenting with typical clinical, biochemical, and radiological indicators of ABPA. In the absence of an asthma history, such patients often undergo investigations directed towards cancer or TB diagnosis, potentially resulting in empiric TB therapy. While acknowledging the potential presence of dual pathologies, such as ABPA coexisting with lung cancer or TB, diagnosing ABPA solely based on meeting the remaining diagnostic criteria, aside from a history of asthma or CF, and with appropriate follow-up, may be more beneficial. This approach not only mitigates patient anxiety and psychological implications regarding potential serious diagnoses but also prudently diminishes the need for a series of invasive investigations and the associated expenses.
